# Primary nodular chest amyloidoma: A case report and review of literature

**DOI:** 10.1016/j.radcr.2021.11.048

**Published:** 2021-12-21

**Authors:** Matthew A. Crain, Georgia M. Vasilakis, Jessica R. Adkins, Ayodele Adelanwa, Jeffery P. Hogg, Dhairya A. Lakhani, Cathy Kim

**Affiliations:** a West Virginia University School of Medicine; b Eberly College of Arts and Sciences, West Virginia University; c Department of Pathology, West Virginia University, Morgantown, WV; d Section of Cardiothoracic Imaging, Department of Radiology, West Virginia University, 1 Medical Center Drive Morgantown, WV 26506, USA

**Keywords:** CT, Computed Tomography, MRI, Magnetic Resonance Imaging, PET, Positive Emission Tomography, Pulmonary amyloidoma, Chest wall mass, Plasmacytoma, Computed Tomography (CT), Magnetic Resonance Imaging (MRI), Positive Emission Tomography (PET)

## Abstract

Primary nodular chest wall amyloidoma, in which a solitary mass of amyloid is deposited in and around the lungs with no evidence of systemic amyloidosis, is extremely rare, most often asymptomatic, and may resemble primary bronchogenic carcinoma. As a result, there are fewer than 100 cases published in the literature and no controlled clinical trials. Primary nodular chest wall amyloidoma is typically diagnosed either as an incidental radiological finding or after very serious and destructive mass growth at which point late-stage respiratory and pain symptoms finally develop, most often in elderly patients. We present imaging studies of a 61-year-old male patient with an unusually massive and destructive chest wall mass, originating in the chest wall, diagnosed as chest wall amyloidoma by histopathology analysis. Our CT, MRI, and PET scan findings are consistent with and contribute to the developing pattern of imaging characteristics seen in other case studies, which can be used to identify amyloidoma before it becomes destructive using non-invasive imaging analyses.

## Background

Amyloidosis is a rare disease, characterized by the deposit of an abnormal insoluble protein, amyloid, in extracellular tissues and organs, resulting in serious dysfunction and potentially death, if untreated [1]. Amyloidosis can be either primary or secondary, and amyloid deposits can be systemic or localized [2]. This disease typically manifests in cardiac, gastrointestinal, renal, hematologic, neurologic, and musculoskeletal tissues and organs [1]. Amyloidosis in the respiratory system involves predominantly amyloid light chain (AL) deposits and can manifest in three forms: tracheobronchial amyloidosis, diffuse alveolar-septal amyloidosis, and nodular parenchymal pulmonary amyloidoma [2]. Tracheobronchial amyloidosis is the most common form of localized primary pulmonary amyloidosis [3]. Localized nodular pulmonary amyloidoma, in which a solitary mass of amyloid is deposited in and around the lungs with no evidence of systemic amyloidosis, is extremely rare, most often asymptomatic, and may resemble primary bronchogenic carcinoma [1,^2^,^4^,5]. As a result, there are fewer than 100 cases published in the literature [1,[4], [5], [6], [7], [8], [9], [10], [11], [12], [13] and no controlled clinical trials.

Primary nodular pulmonary amyloidoma is typically diagnosed either as an incidental finding or after very serious and destructive mass growth at which point late-stage respiratory and pain symptoms finally develop, most often in elderly patients [1,2]. Diagnosis of nodular pulmonary amyloidoma is also challenging since the presenting symptoms, when they occur, as well as routine lab tests and imaging studies often mimic more common diseases, such as infectious, inflammatory, or neoplastic conditions, including primary lung carcinoma [2,5]. While the gold standard for the diagnosis of amyloidosis is congo red staining of biopsy tissue, this procedure is invasive and has intrinsic risk, especially within the respiratory system [1,2]. Therefore, there is a need for clarification of radiologic imaging characteristics, which could provide a less invasive approach to diagnosis. Toward this end, we present imaging studies of a patient with an unusually massive and destructive chest wall mass, diagnosed as primary nodular chest wall amyloidoma by histopathology analysis.

## Case report

A 61-year-old male was transferred from an outside facility for worsening shortness of breath while performing yard work over the prior three weeks. The patient had a past medical history of hypertension, congestive heart failure, severe chronic obstructive pulmonary disease (COPD), smoking (until age 46), and a right apical lung mass confirmed to be a plasmacytoma (at age 50). Upon arrival at our facility, the patient was afebrile with heart rate of 76 and blood pressure of 156/92. Routine labs including complete blood count with differential as well as basic metabolic and liver function panels were unremarkable. His venous blood gas revealed a mild respiratory alkalosis. COVID-19 test was negative. The patient was admitted for further evaluation.

**Fig. 1:**
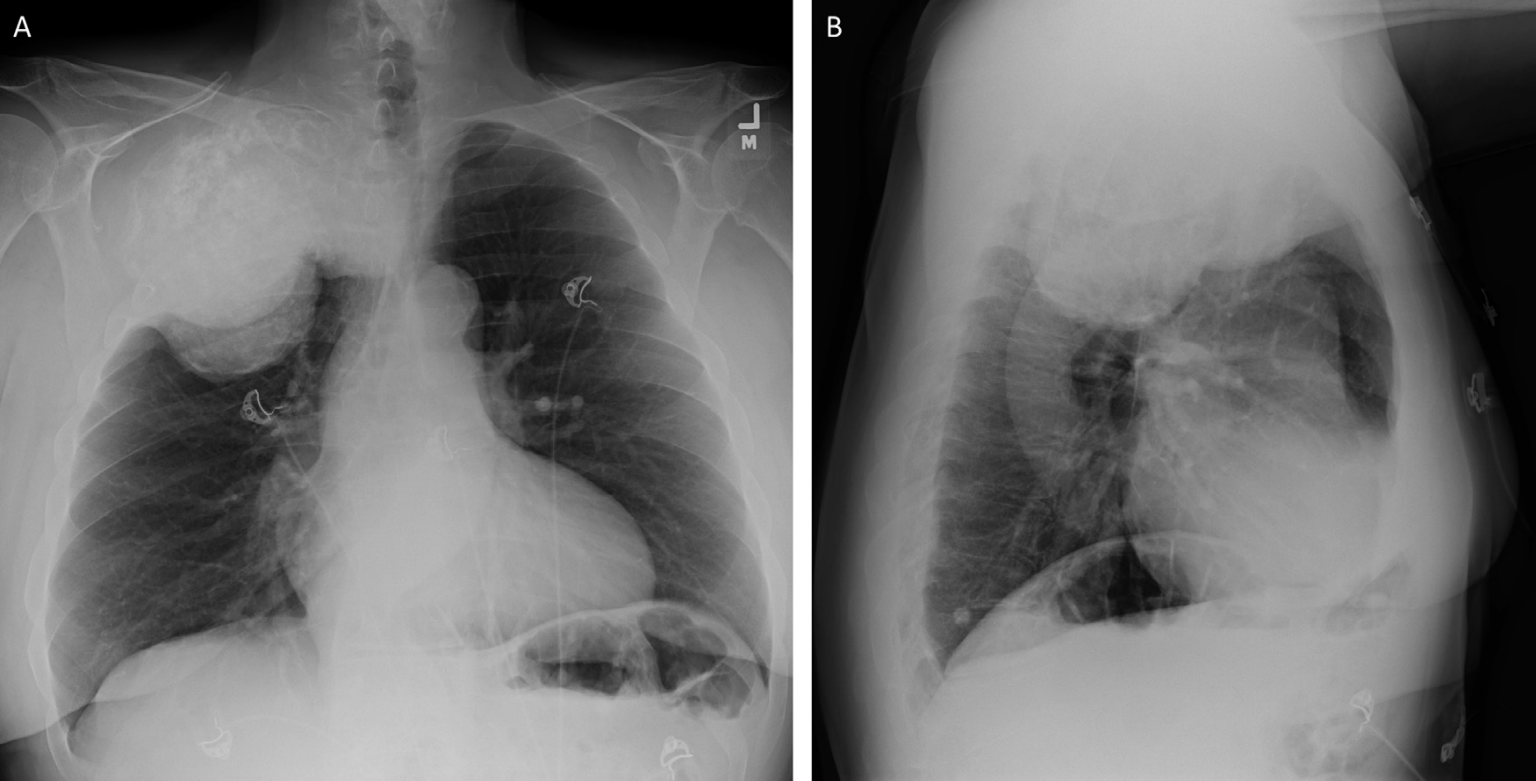
AP (A) and lateral projection (B) radiographs demonstrate a large right apical chest wall mass. This mass demonstrates foci of calcifications

**Fig. 2:**
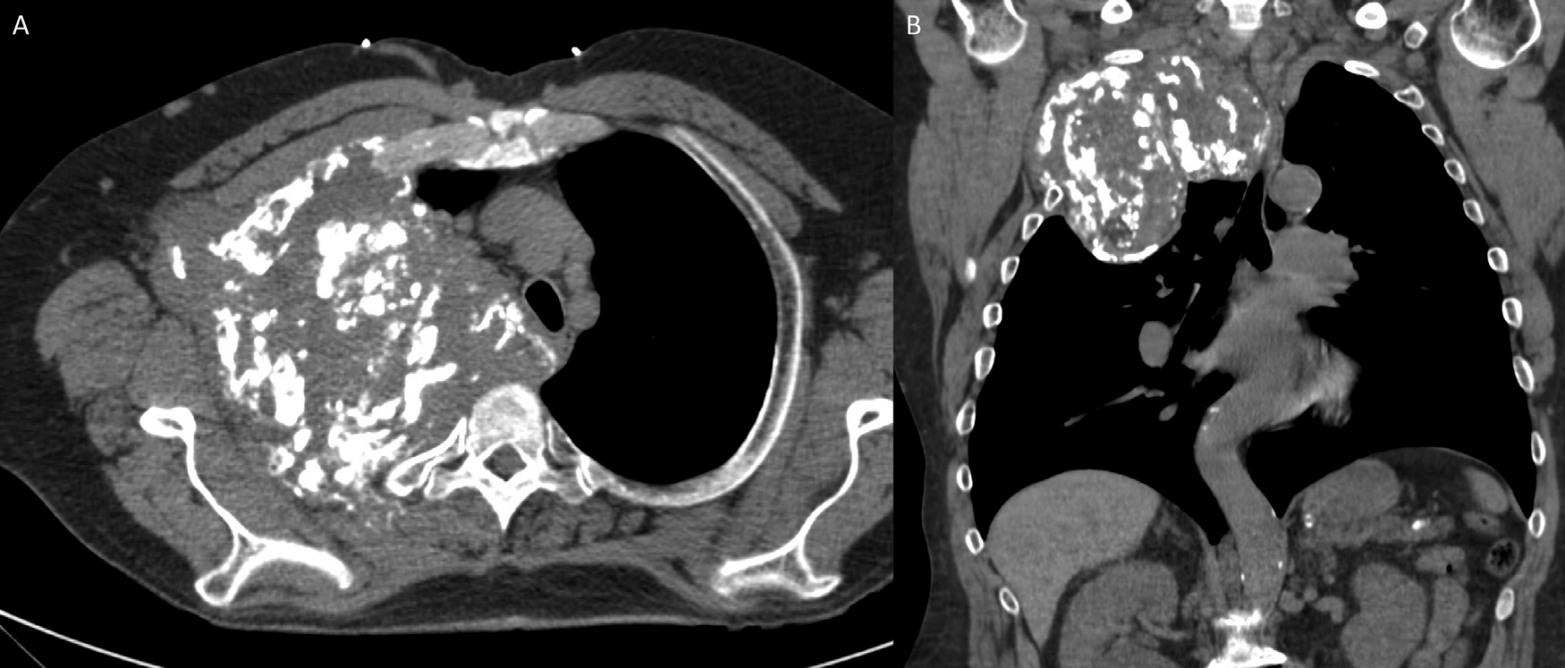
Unenhanced CT of the chest, axial (A) and coronal (B) demonstrates a solid soft tissue mass in the right upper chest wall measuring 13.3 × 15.6 × 13.4 cm, with prominent amorphous internal foci of calcifications. This mass resulted in mass effect on the trachea and superior mediastinal structures. This mass extends through the chest wall into the right axilla, and erodes the right second rib

**Fig. 3:**
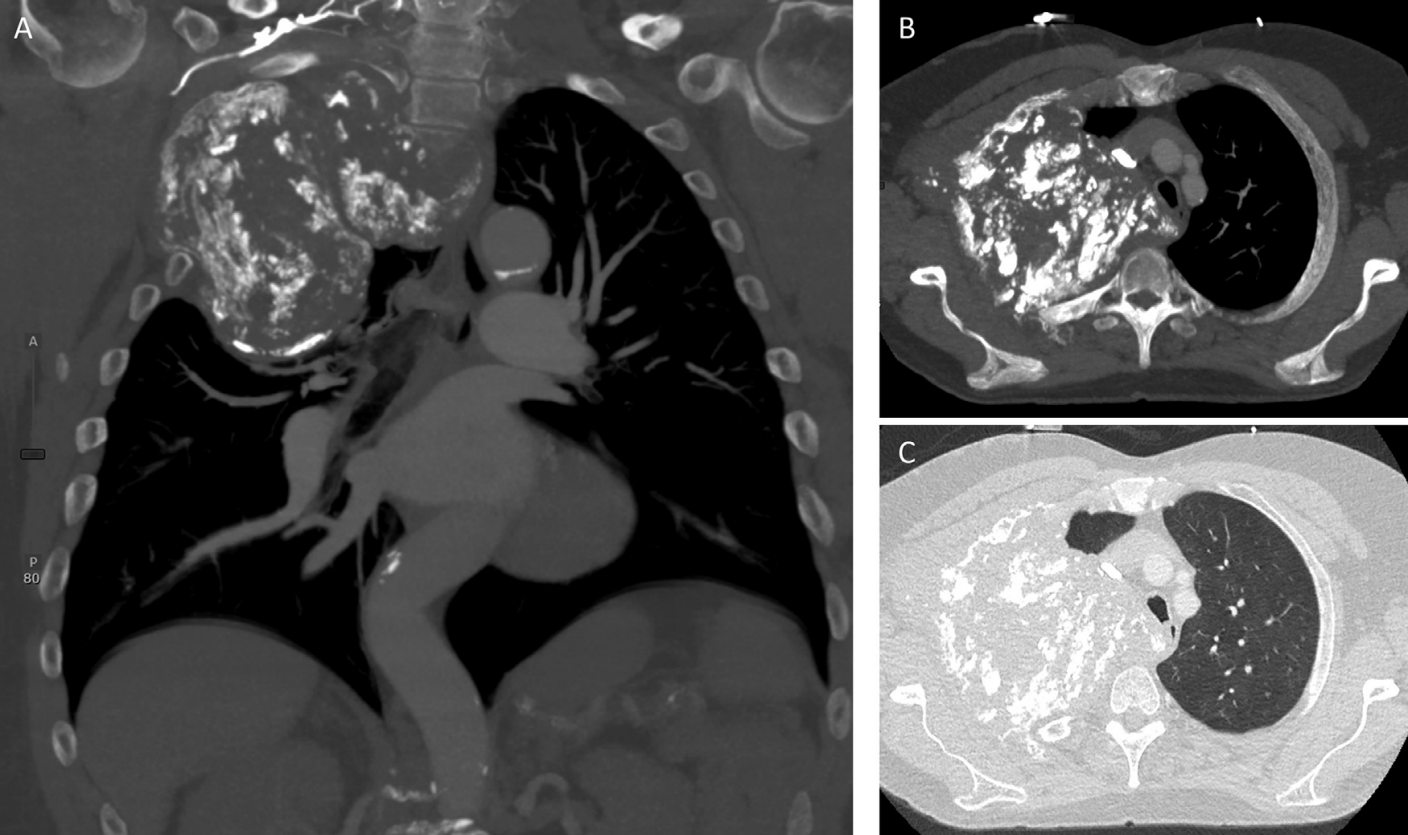
Contrast-enhanced CT of the chest, coronal (A), mediastinal window axial (B) and lung window axial (C) demonstrates no abnormal enhancement in right upper chest wall mass that extends through the pleura into the right axilla and it erodes the right second rib

Radiographs demonstrated a large right apical lung mass with foci of intramural calcifications (Fig. 1). Computed tomography (CT) chest revealed a large expansile destructive mass arising from the right second rib, measuring 15 cm x 13.5 cm x 12.6 cm (Fig. 2). The mass invaded the right chest wall, the mediastinum, and the T2 andT3 vertebrae, causing tracheal displacement and narrowing. The mass had calcifications and a multilobular appearance (Fig. 3).

**Fig. 4:**
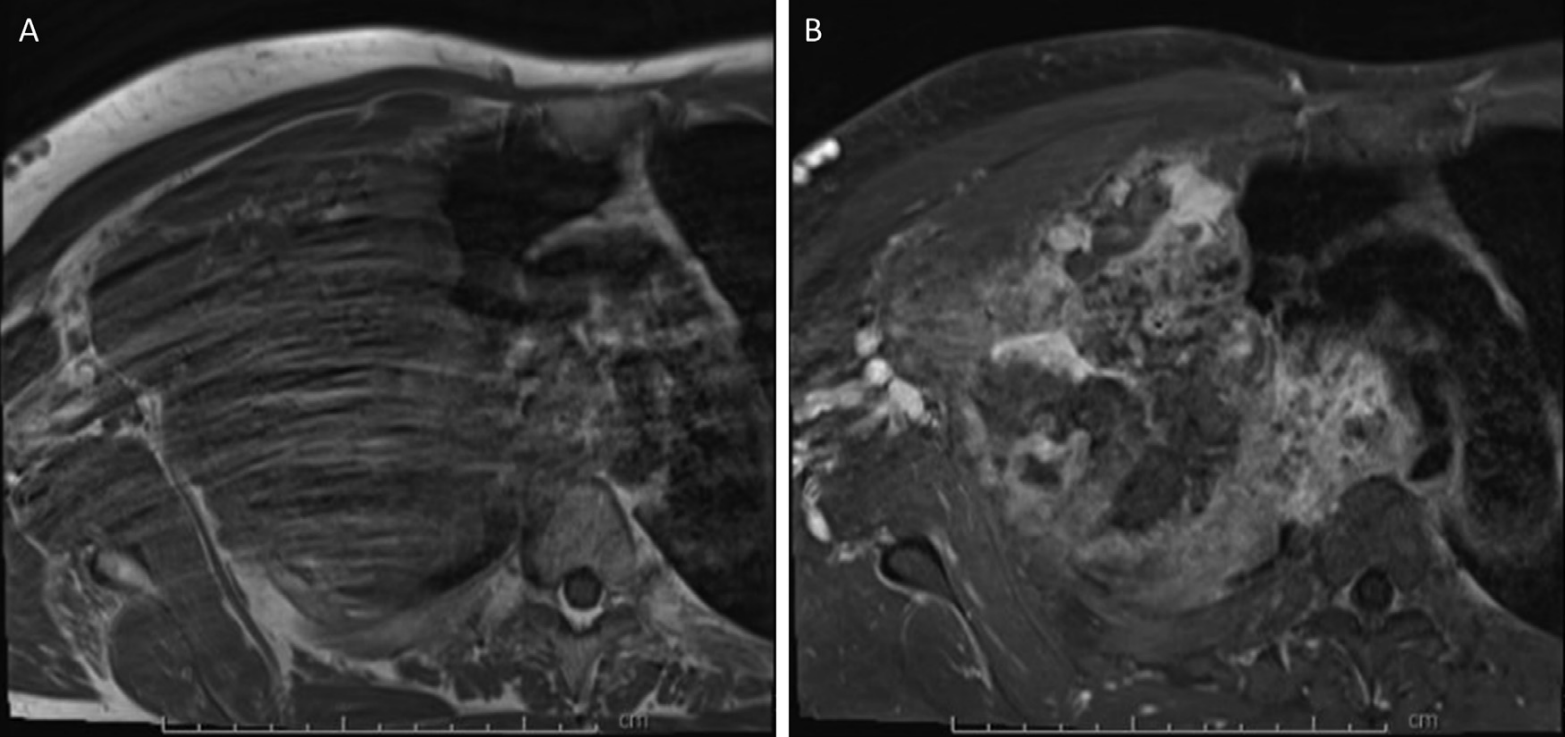
MRI brachial plexus protocol, T1 axial (A), and T1 post gadolinium (B) demonstrates a T1 isointense peripherally enhancing right upper chest wall mass extending into the right axilla, and eroding right second rib

**Fig. 5:**
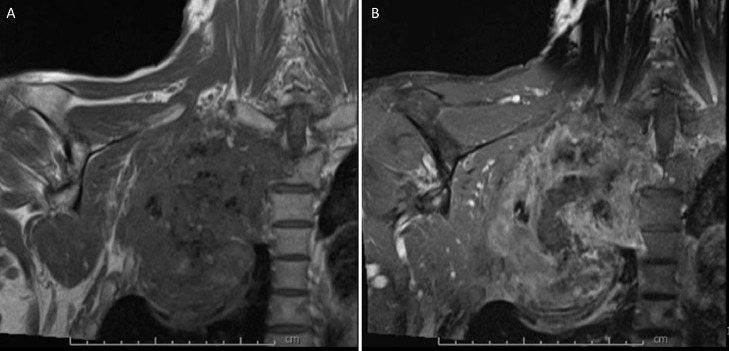
MRI brachial plexus protocol, T1 coronal (A), and T1 coronal post gadolinium (B) demonstrates a T1 isointense peripherally enhancing right upper chest wall mass extending into the right axilla, and eroding right second rib

**Fig. 6:**
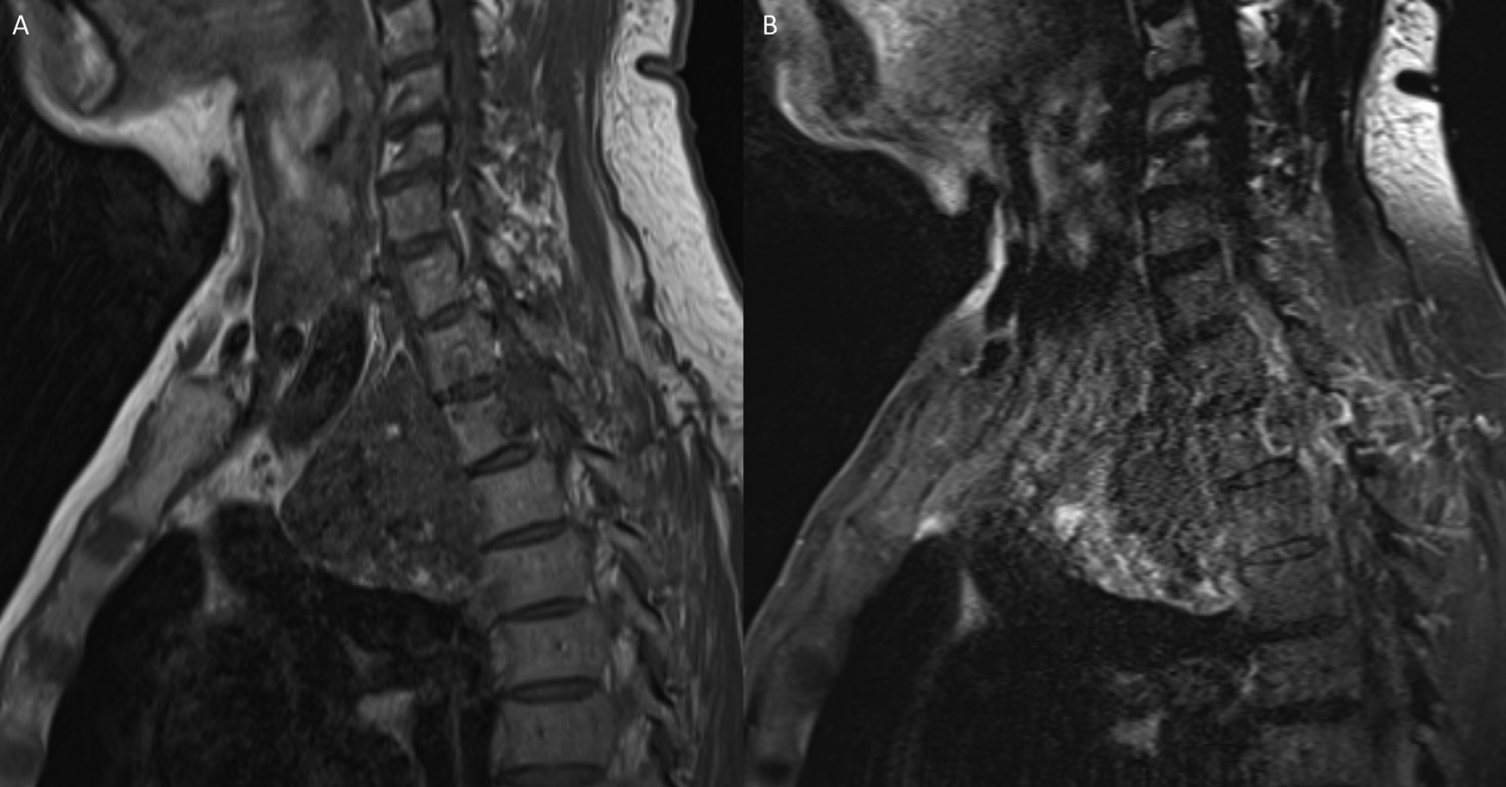
MRI brachial plexus protocol, T1 coronal (A) and T1 post gadolinium coronal (B) demonstrates extension of this mass into the right T1-T2 and T2-T3 neural foramina (not included in the figure) and T1-T2 right lateral epidural space, with mild mass effect on the thecal sac

Magnetic resonance imaging (MRI) of the right brachial plexus (Fig. 4, Fig. 5, Fig. 6) without and with IV contrast was obtained to evaluate for local invasion. MRI analyses revealed a heterogeneously enhancing and ossified mass that destroyed the right second rib, the right T2 transverse process, pedicle, articular pillar, and lateral aspect of the right T2 vertebral body (Figs. 4-6). The tumor extended into the right T1-T2 and T2-T3 neural foramina and entered the epidural space in the right lateral aspect of the T1-T3 vertebrae, which caused a mild mass effect on the thecal sac (Figs. 4-6). The mass invaded the chest wall and extended into the right axillary region and superior mediastinum. The mass also abutted and displaced the esophagus, trachea, superior vena cava, and brachiocephalic artery to the left. Tumor involvement of the right T1 nerve was noted, but the majority of the right brachial plexus was spared (Figs. 4-6). No suspicious cervical lymphadenopathy or marrow-replacing lesions were noted. Fat planes in the neck were preserved without tumor invasion. PET/CT scan revealed a large partially calcified right chest wall mass extending across the pleura into the axilla, which displayed hypermetabolic activity with max SUV 5.44. Lytic lesions were seen at the right ischium and the right vertebral body of T10.

**Fig. 7:**
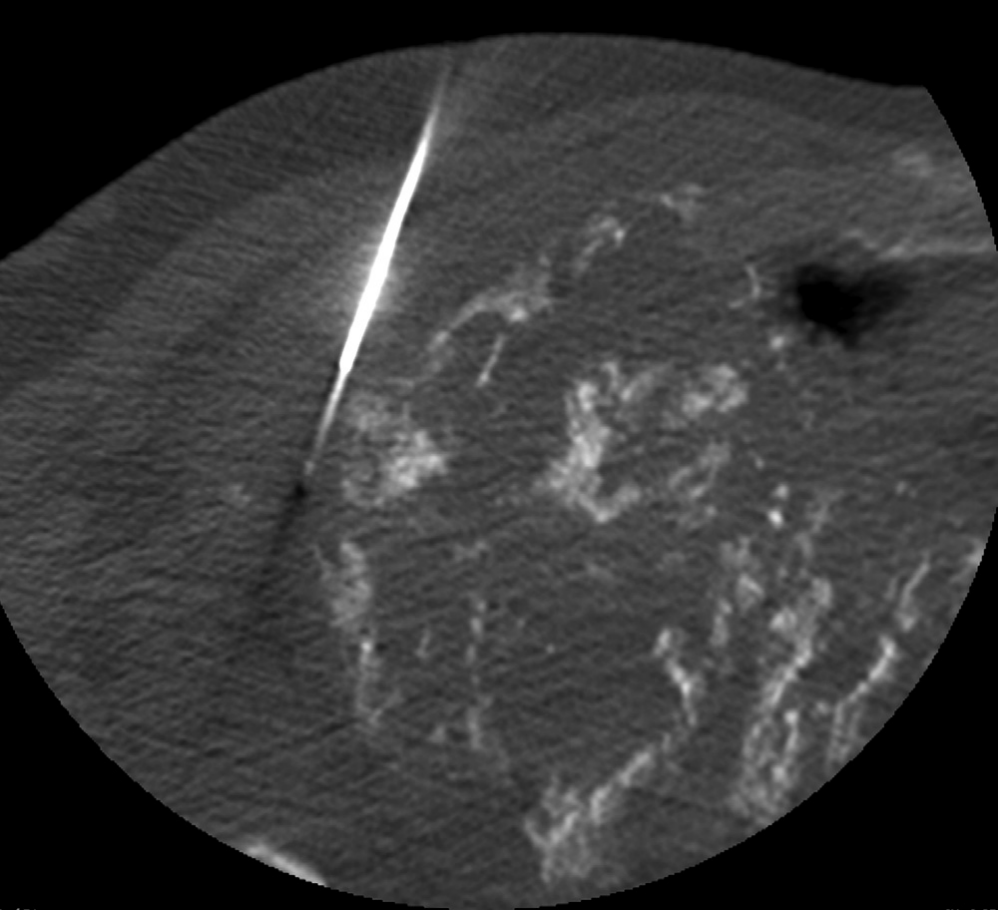
CT-guided 16-gauge core biopsy of right upper lobe lung mass soft tissue component was obtained

**Fig. 8:**
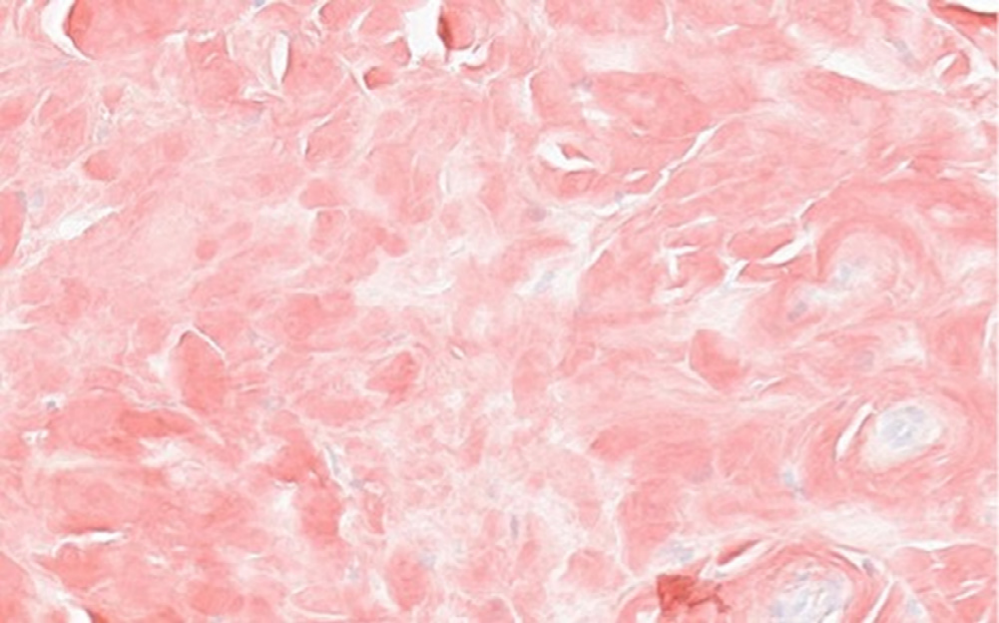
Section of pulmonary biopsy showing abundant eosinophilic amorphous material with apple-green birefringence consistent with amyloid deposition. Congo red stain (400x)

Differential diagnoses of the chest wall mass, based upon imaging studies, included plasma cell neoplasm (benign or malignant), plasmacytoma, or amyloidoma. An image-guided biopsy was performed to clarify the diagnosis. Multiple 16-gauge core biopsies of the soft tissue component of the right lung mass were obtained (Fig. 7). Pathology report revealed abundant eosinophilic amorphous material with rare embedded fine strands of fibrous tissue with apple-green birefringence, which was diagnosed as amyloidoma (Fig. 8), leading to a diagnosis of primary nodular chest wall amyloidoma.

Image-guided cardiothoracic and neurosurgical resections were performed to reduce the impact of the mass on thoracic structures including right-sided T1-T3 hemilaminectomies and facetectomies. Pathology confirmed the diagnosis of amyloidoma. The surgeries were successful and the patient was discharged to outpatient follow-up care. Our last medical contact with the patient was two months after surgery, when he tested positive for COVID-19. Follow-up two months later determined that he recovered from COVID-19 and is doing well.

## Discussion

The term amyloid was first used by Rudolf Virchow in 1854 [14]. Amyloidosis is a heterogeneous spectrum of diseases characterized by the deposition of misfolded insoluble proteins in extracellular tissue. Amyloidosis can be primary or secondary, and the deposition of amyloid proteins can be localized or systemic. Localized pulmonary amyloidoma was first described by Lesser in 1877 [15], and has been identified in three main anatomical areas: tracheobronchial, parenchymal, and mediastinal [16].

Diagnosis of localized pulmonary amyloidoma is extremely rare, in part due to the unusual nature of the condition, but also because the patients are often asymptomatic and the mass is very slow-growing. While respiratory symptoms, such as cough, dyspnea, and hemoptysis are seen in certain forms of localized pulmonary amyloidosis, such as tracheobronchial, which facilitates detection [3], most patients with primary nodular pulmonary amyloidosis are unaware of the growth of the mass in their lung. As a result, the mass can become extremely large and destructive before it comes to medical attention, and the patients are typically older than in other forms of amyloidosis [2]. As far as we know, our patient's pulmonary amyloidoma mass, which originated in the chest wall, was among the most massive and destructive to adjoining tissue, organs, and bones on record. The diagnosis was also complicated since the presenting symptoms, notably shortness of breath, were also consistent with the patient's known medical conditions including COPD and could have represented a COPD exacerbation.

Only one case with a clinical course similar to our patient has been reported recently by Desai et al [1]. Desai and his colleagues treated that first reported case of a patient with severe shortness of breath necessitating hospital admission due to a very large solitary pulmonary nodular amyloidoma [1]. As with our patient, the pulmonary mass grew asymptomatically for a long period, so it was much larger and more destructive to adjoining organs, tissues, and bones than most other amyloidoma anatomical locations. CT chest scan showed a lobulated heterogeneous mass with adjacent lytic lesion, similar to our patient.

There have only been a few published case reports of patients with pulmonary amyloidoma and plasmacytoma [17], [18], [19], [20], reflecting the extreme rarity of both of these unusual conditions in our patient. The first case, published in 2012 was of a patient with pulmonary plasmacytoma who was then diagnosed a decade later with a large pulmonary amyloidoma nodular mass [17], similar to our patient's progression. Shortly after, three cases of concurrent solitary plasmacytoma and nodular pulmonary amyloidoma were published [18], [19], [20]. While further investigation of the possible etiological relationship between these two conditions is recommended, the diagnosis of pulmonary plasmacytoma should alert physicians to regularly assess for the potential development of pulmonary nodules or masses, including amyloidoma, to prevent the severe growth and destruction, as seen in our patient.

Therefore, regular imaging studies are suggested, especially for patients with presenting respiratory symptoms, such as shortness of breath, cough, dyspnea, and hemoptysis, as well as known pulmonary conditions including COPD, plasmacytoma, and amyloidosis, in order to identify the development of expansile and destructive pulmonary nodes. Primary nodular pulmonary amyloidoma mimics a variety of serious lung diseases including malignant tumors as well as interstitial, granulomatous or inflammatory respiratory diseases [11]. Although pulmonary amyloidoma is a rare disease, it needs to be considered in the differential diagnosis. While the gold standard for the diagnosis of amyloidosis is histopathological assessment by congo red staining, biopsy is invasive and has inherent risks, especially involving the respiratory system [2]. Therefore, prior to pulmonary biopsy, imaging studies have a critical role in the non-invasive assessment and diagnosis of respiratory disease as well as the identification and characterization of soft tissue tumors. At present, diagnosis of primary pulmonary nodular amyloidoma based on imaging studies is challenging, but may become more informative as algorithms are established based on accumulating data regarding imaging characteristics.

Case reports in the literature suggest that preliminary evidence of primary nodular pulmonary amyloidoma may be revealed as incidental findings on chest radiographs, though differential diagnosis is limited [2]. Specific CT scan features have been described in published case reports [1,^2^,[4], [5], [6],^11^,12], and provide certain patterns that may facilitate preliminary evidence of nodular pulmonary amyloidoma. One of the first case reports that described CT features of nodular pulmonary amyloidoma was published by Matsumoto et al., which revealed small calcifications and slightly irregular margins [11]. Kim et al. presented a general description of CT scans of nodular pulmonary amyloidoma, characterized by a pattern of tiny to massive nodules with sharp and lobulated margins and with calcifications in about half of the patients, similar to our patient [12].

In the most recent and detailed analysis of CT chest scans in 41 patients with nodular pulmonary amyloidoma, Brandelik et al. found that all patients presented predominately with a nodular parenchymal pattern, with 39 of them exhibiting solitary or multiple pulmonary nodules [6]. Most of the patients had lesions with a smooth or lobulated border, with calcifications in the lesions in about half of their patients, similar to our patient.

Only a few studies have described the MRI findings in patients with primary pulmonary amyloidoma [1,11]. Matsumoto et al. reported that the pulmonary mass appeared isointense relative to skeletal muscle on T1-weighted axial MRI, but slightly lower intensity on T2-weighted images [11]. MRI analyses have included a heterogeneously enhancing mass [1,4] and unusual cystic radiological features [2]. Desai et al. recently reported highly variable MRI signal intensities in the diagnosis of amyloidomas,[1] similar to the findings in our patient.

Several cases have been published with PET/CT scan findings for solitary nodular pulmonary amyloidoma. One of the first case reports showed increased metabolic activity of the calcified mass with numerous osseous lytic lesions [17]. Case reports have reported PET/CT scans that showed metabolically active tissue with morphological characteristics of a possible neoplastic process [5]. More recently, PET/CT scan findings reported for amyloidomas have found pathologically elevated utilization of the glucose analog, and one specifically in a patient with solitary pulmonary amyloidoma (SUV max 5.25), [5] remarkably similar to our patient.

In summary, imaging features that may suggest primary nodular amyloidoma include the following characteristics, which may be useful in differentiating it from bronchogenic carcinomas, other tumors, and infectious or inflammatory diseases:

(1) Unusually large solitary destructive mass in and around the lungs that is not detected until the patient is elderly, in part, due to slow asymptomatic growth over the years, most often initially identified as an incidental finding on a radiograph.
(2) CT scans have generally shown lobulated margins with calcifications in a heterogeneously enhancing mass and unusual cystic radiological features.
(3) MRI analyses generally demonstrate heterogeneously enhancing mass with highly variable signal intensities; specifically, the lung mass may appear isointense relative to skeletal muscle on T1-weighted axial MRI, but relatively low intensity on T2-weighted MRI.
(4) PET/CT scan may show pathological utilization of the glucose analog and hypermetabolic activity with unusually high SUV, along with lytic lesions.

While the diagnosis of slow-growing asymptomatic pulmonary nodular amyloidoma can be challenging, we have provided evidence that routine imaging studies, using our expanding knowledge of specific imaging patterns, may be useful to identify growing masses before they become destructive as well as avoid unnecessary invasive procedures. Once imaging studies are suggestive of amyloidoma, pathohistological diagnostic confirmation is still required by a pulmonary biopsy and demonstration of apple-green birefringence with congo red stain under polarized light.

Excision of the pulmonary mass is often recommended in the management of these patients, especially for large amyloidoma lesions, in order to reduce the destructive impact on adjoining organs, tissue, and bones as well as to improve respiratory functioning. Radiotherapy can be a viable option for the treatment of solitary pulmonary amyloidoma [2]. Prognosis is usually fairly good due to its localized nature, except when the surrounding regions become severely damaged.

Primary pulmonary nodular amyloidoma is an extremely rare diagnosis. Our case report of an unusually massive chest wall amyloidoma, with a history of plasmacytoma, contributes to the accumulation of specific imaging features regarding diagnosis of rare pulmonary diseases as differentiated from more commonly seen disorders, which is critical in developing appropriate treatment plans and prognostic guidance. Early imaging detection is critical in order to avoid serious displacement and destruction of adjoining organs, tissues, and bones.

## Patient consent

Written informed consent was obtained for this Case Report including publication of imaging studies.
